# *YUCCA*-mediated auxin biogenesis is required for cell fate transition occurring during *de novo* root organogenesis in Arabidopsis

**DOI:** 10.1093/jxb/erw213

**Published:** 2016-06-02

**Authors:** Lyuqin Chen, Jianhua Tong, Langtao Xiao, Ying Ruan, Jingchun Liu, Minhuan Zeng, Hai Huang, Jia-Wei Wang, Lin Xu

**Affiliations:** ^1^National Key Laboratory of Plant Molecular Genetics, CAS Center for Excellence in Molecular Plant Sciences, Institute of Plant Physiology and Ecology, Shanghai Institutes for Biological Sciences, Chinese Academy of Sciences, 300 Fenglin Road, Shanghai 200032, China; ^2^University of Chinese Academy of Sciences, 19A Yuquan Road, Beijing 100049, China; ^3^Pre-National Laboratory for Crop Germplasm Innovation and Resource Utilization, Hunan Agricultural University, Changsha, Hunan 410128, China; ^4^College of Life and Environment Sciences, Shanghai Normal University, Shanghai 200234, China

**Keywords:** *Arabidopsis*, auxin biogenesis, *de novo* root organogenesis, plant regeneration, *WOX11*, *YUCCA.*

## Abstract

Many plant organs have the ability to regenerate a new plant after detachment or wounding via *de novo* organogenesis. During *de novo* root organogenesis from *Arabidopsis thaliana* leaf explants, endogenic auxin is essential for the fate transition of regeneration-competent cells to become root founder cells via activation of *WUSCHEL-RELATED HOMEOBOX 11* (*WOX11*). However, the molecular events from leaf explant detachment to auxin-mediated cell fate transition are poorly understood. In this study, we used an assay to determine the concentration of indole-3-acetic acid (IAA) to provide direct evidence that auxin is produced after leaf explant detachment, a process that involves *YUCCA* (*YUC*)-mediated auxin biogenesis. Inhibition of YUC prevents expression of *WOX11* and fate transition of competent cells, resulting in the blocking of rooting. Further analysis showed that *YUC1* and *YUC4* act quickly (within 4 hours) in response to wounding after detachment in both light and dark conditions and promote auxin biogenesis in both mesophyll and competent cells, whereas *YUC5*, *YUC8*, and *YUC9* primarily respond in dark conditions. In addition, *YUC2* and *YUC6* contribute to rooting by providing a basal auxin level in the leaf. Overall, our study indicates that *YUC* genes exhibit a division of labour during *de novo* root organogenesis from leaf explants in response to multiple signals.

## Highlight

YUCCA family genes act in response to multiple signals in leaf explants and contribute to *de novo* auxin biogenesis for fate transition of regeneration-competent cells.

## Introduction

Plants have powerful abilities to regenerate. Many detached or wounded plant organs can undergo *de novo* organogenesis—the regeneration of adventitious shoots and roots upon wounding—allowing the individual to survive various levels of damage (Duclercq *et al.*, 2011; Sugimoto *et al.*, 2011; Xu and Huang, 2014). The natural ability of plants to spontaneously regenerate has also been exploited for tissue culture and is widely used in modern agriculture (Sussex, 2008).

In a previous study, we used a simple method to examine *de novo* root organogenesis by culturing *Arabidopsis thaliana* leaf explants on B5 medium without added exogenous hormones: our results suggested that two steps are involved in cell fate transition during the regeneration of adventitious roots (Chen *et al.*, 2014; Liu *et al.*, 2014). Activation of *WUSCHEL-RELATED HOMEOBOX 11* (*WOX11*) is involved in the first step of cell fate transition from regeneration-competent cells (i.e. procambium and vascular parenchyma cells) to root founder cells. Cell division occurs in the second step of cell fate transition as root founder cells transition into becoming root primordium cells, which is marked by *WOX5*. Finally, adventitious roots are differentiated from the root primordium (Liu *et al.*, 2014).

Auxin is a critical hormone that controls *de novo* root organogenesis (Greenwood *et al.*, 2001; De Klerk, 2002; Ahkami *et al.*, 2009; Gutierrez *et al.* 2009; Correa Lda *et al.*, 2012; Sukumar *et al.* 2013; Liu *et al.*, 2014; Xu and Huang, 2014). In this rooting system, we previously observed that endogenous auxin is essential for cell fate transition because blocking auxin polar transport resulted in the loss of the auxin maximum in competent cells near the wound and thus blocked regeneration (Liu *et al.*, 2014). The auxin maximum in competent cells was assumed to directly trigger expression of *WOX11*, because mutations of the auxin response elements on the *WOX11* promoter resulted in partial blocking of its expression in competent cells (Liu *et al.*, 2014).

The behaviour of auxin seems to be activated after detachment of the leaf from the source plant during the early stage of adventitious root regeneration from leaf explants (Liu *et al.*, 2014). However, the molecular events between leaf detachment and auxin-mediated cell fate transition are barely known. Thus, the important question is how endogenous auxin accumulates in leaf explants after detachment.

Auxin biosynthesis is involved in multiple developmental processes (Chandler, 2009). The main natural auxin, indole-3-acetic acid (IAA), is biosynthesized primarily via two chemical reactions (Zhao, 2012). First, the amino acid tryptophan (Trp) is catalysed by the TAA/SAC family of tryptophan aminotransferases to become indole-3-puruvate (IPA) (Stepanova *et al.*, 2008; Tao *et al.*, 2008). Next, IPA is converted to IAA by the catalysis of flavin-containing monooxygenases encoded by *YUCCA* (*YUC*) genes, and this is a rate-limiting step (Zhao *et al.*, 2001; Mashiguchi *et al.*, 2011; Won *et al.*, 2011). In this study, we provide insight into the multiple roles of *YUC*-mediated endogenous auxin biosynthesis during *de novo* root organogenesis.

## Materials and methods

### Plant materials and culture conditions

Wild-type *Arabidopsis thaliana* Columbia-0 (Col-0) was used in this study. *yuc* mutants were in the Col-0 background and have been previously described (Cheng *et al.*, 2006; Robert *et al.*, 2013). The *DR5*_*pro*_ *:GUS* line in Landsberg *erecta* (Ulmasov *et al.*, 1997) and the *WOX11*_*pro*_ *:GUS*, *WOX5*_*pro*_ *:GUS*, and *35S*_*pro*_ *:WOX11* lines in Col-0 (Liu *et al.*, 2014) have also been described previously.

Our previous protocols describe the growth conditions of plants and culture conditions of leaf explants for *de novo* root organogenesis in either light or dark conditions (Chen *et al.*, 2014). All leaf explants used in this study were from the first pair of rosette leaves from 12-day-old seedlings, except those from 15-day-old seedlings that were used for thin sectioning. Yucasin (cat. 573760, Sigma, USA) treatment was carried out as previously described (Nishimura *et al.*, 2014).

*YUC4*_*pro*_ *:GUS*, *YUC1*_*pro*_ *:GUS*, *YUC2*_*pro*_ *:GUS*, and *YUC9*_*pro*_ *:GUS* were constructed by insertion of a 3.7-kb promoter of *YUC4*, a 3.6-kb promoter of *YUC1*, a 3.0-kb promoter of *YUC2*, and a 4.0-kb promoter of *YUC9* into the pBI101 vector, respectively. These constructs were verified by sequencing and were introduced into wild-type Col-0 plants by *Agrobacterium*-mediated transformation. Primers for cloning are listed in Supplementary Table S1 at *JXB* online.

### Determination of IAA concentrations

The first pair of rosette leaves of 12-day-old seedlings at 0, 4, and 12 hours after culture (HAC) on B5 medium in dark or light conditions were harvested and frozen in liquid nitrogen; each sample contained ~160–170 fresh leaves. The [^2^H_5_]-IAA measurement was carried out as previously described (Zhang *et al.*, 2014).

### GUS staining, reverse transcription (RT)-PCR, quantitative RT-PCR, and ChIP

GUS staining was performed as previously described (Chen *et al.*, 2014; Liu *et al.*, 2014) and observed using Nikon Eclipse 80i and Nikon SMZ1500 microscopes (Nikon, Japan).

For reverse transcription (RT)-PCR and quantitative (q)RT-PCR, RNA extraction and reverse transcription were performed as described previously (He *et al.*, 2012). The qRT-PCR results represented the relative expression levels, which were normalized against those produced by the primers for *ACTIN*, whose values were arbitrarily fixed at 1.0.

ChIP was performed as previously described (He *et al.*, 2012) using the antibody against of histone H3 lysine 27 trimethylation (H3K27me3; cat. 07-449, Millipore, USA). The ChIP results were normalized against those produced by the primers for *AG*, and the values of time-0 leaf explants were arbitrarily fixed at 1.0. Primers for RT-PCR, qRT-PCR, and ChIP are listed in Supplementary Table S1.

## Results

### Auxin production during *de novo* root organogenesis

**Fig. 1.**
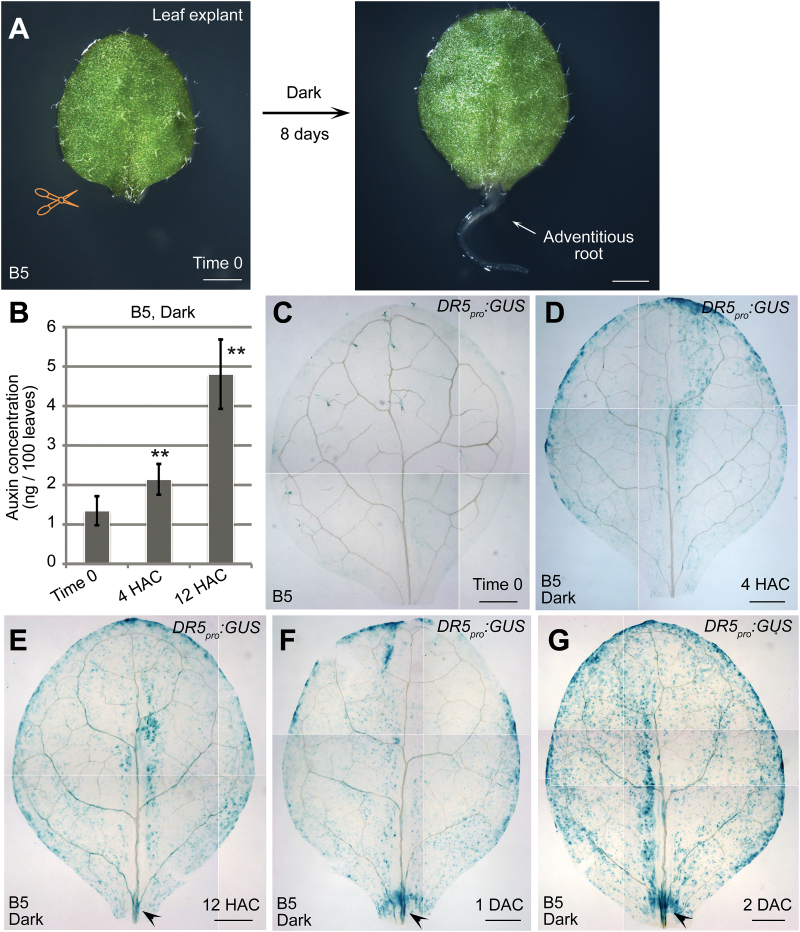
Auxin production in leaf explants after detachment. (**A**) System of *de novo* root organogenesis on B5 medium with sucrose in darkness. (**B**) Auxin concentration in leaf explants from time 0 to 12 HAC on B5 medium. Bars show SEM with three biological replicates. Each biological replicate was performed with three technical replicates. **P < 0.01 in two-sample *t* tests compared with time-0 control. (**C–G**) Observations of the GUS signal in leaf explants from *DR5*_*pro*_ *:GUS* reporter line at time 0 (C), 4 HAC (D), 12 HAC (E), 1 DAC (F), and 2 DAC (G). Arrowheads in E–G indicate the GUS signal in vasculature near the wound. The data in C–G were pasted together from small pictures of the same leaf explant because the microscope was unable to capture the entire leaf explant in a single visual field. Scale bars, 1mm in A and 500 μm in C–G.

To study the early events after detachment of leaf explants, we cultured leaf explants on B5 medium without added hormones in dark conditions (Fig. 1A) (Chen *et al.*, 2014). We previously showed that endogenous auxin plays important role in the regeneration of adventitious roots (Liu *et al.*, 2014). To directly test the auxin levels in leaf explants during regeneration, we performed an assay to determine the IAA concentration. The results showed that the level of auxin was gradually and significantly elevated in leaf explants within 12 HAC on B5 medium compared with those prior to culture (time 0) (Fig. 1B), indicating that auxin is quickly produced after the detachment of leaf explants from source plants.

To further observe the spatiotemporal pattern of auxin production, we conducted the analysis using the *DR5*_*pro*_ *:GUS* reporter line, which reflects the auxin level by monitoring auxin responsiveness (Ulmasov *et al.*, 1997). Our data showed that the GUS signal quickly became elevated in mesophyll cells of leaf explants within 4 HAC (Fig. 1C, D). The GUS signal then gradually increased and began to accumulate in the vasculature near the wound at 12 HAC (Fig. 1E). The GUS signal continued to concentrate in the vasculature near the wound at 1–2 days after culture (DAC) (Fig. 1F, G) (Liu *et al.*, 2014). Therefore, auxin levels rose quickly in mesophyll cells after detachment of leaf explants, and the auxin was then polar transported into the competent cells in the vasculature near the wound, where it triggers cell fate transition (Liu *et al.*, 2014).

### Identification of *YUC4* and *YUC1* in regeneration

**Fig. 2.**
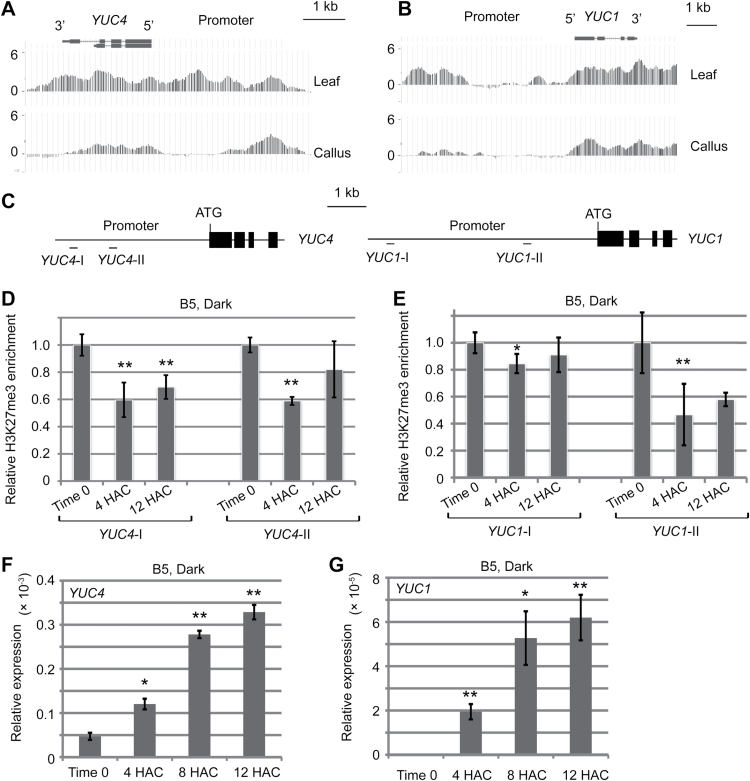
Identification of *YUC4* and *YUC1* in regeneration. (**A, B**) Levels of epigenetic marker H3K27me3 at *YUC4* (A) and *YUC1* (B) loci were reduced in 20-DAC leaf explants that produced callus on callus-inducing medium compared with levels in the time-0 leaf explants. The original ChIP-chip data are from our previous study (He *et al.*, 2012). (**C**) Diagram of *YUC4* and *YUC1* loci with primer positions (*YUC4*-I, *YUC4*-II, *YUC1*-I, and *YUC1*-II) in ChIP analysis in D and E. (**D, E**) ChIP analysis of the H3K27me3 levels at *YUC4* (D) and *YUC1* (E) loci in time-0, 4-HAC, and 12-HAC leaf explants of wild-type Col-0 cultured on B5 medium. (**F, G**) qRT-PCR analysis of expression levels of *YUC4* (F) and *YUC1* (G) within 12 HAC on B5 medium. Bars show SEM with three biological replicates in D–G. Each biological replicate was performed with three technical replicates. *P < 0.05 and **P < 0.01 in two-sample *t* tests compared with time-0 control.

Our previous study showed that *de novo* root organogenesis shares a similar genetic pathway with callus formation (Liu *et al.*, 2014). To reveal the mechanism of auxin behaviour in leaf explants, we therefore searched our previous ChIP-chip data of histone H3K27me3, a marker of the repressive chromatin state of gene expression (Schatlowski *et al.*, 2008; He *et al.*, 2013), during the leaf-to-callus transition on callus-inducing medium (He *et al.*, 2012). We noted that the H3K27me3 levels at the *YUC4* and *YUC1* loci were reduced during the leaf-to-callus transition (Fig. 2A, B) (He *et al.*, 2012). This implies that *YUC* expression is upregulated during regeneration and this upregulation is associated with H3K27 demethylation.

To test this hypothesis, we first measured the H3K27me3 levels at *YUC4* and *YUC1* loci during *de novo* root organogenesis from leaf explants on B5 medium. Our results showed that H3K27me3 levels at *YUC4* and *YUC1* loci were reduced in 4-HAC and 12-HAC leaf explants compared with those at time 0 (Fig. 2C–E), indicating that the two *YUC* genes may also be activated in *de novo* root organogenesis.

qRT-PCR results showed that expression of *YUC4* was at a low level in time-0 leaf explants, became elevated at 4 HAC, and continued to increase at 8 and 12 HAC during culture on B5 medium (Fig. 2F). *YUC1* expression was barely detected in time-0 leaf explants, and gradually upregulated in leaf explants on B5 medium from 4 to 12 HAC (Fig. 2G). Hence, we suppose that the two *YUC* genes are responsible for endogenous auxin production in leaf explants.

### *YUC*-mediated auxin biosynthesis is involved in *de novo* root organogenesis from leaf explants

**Fig. 3.**
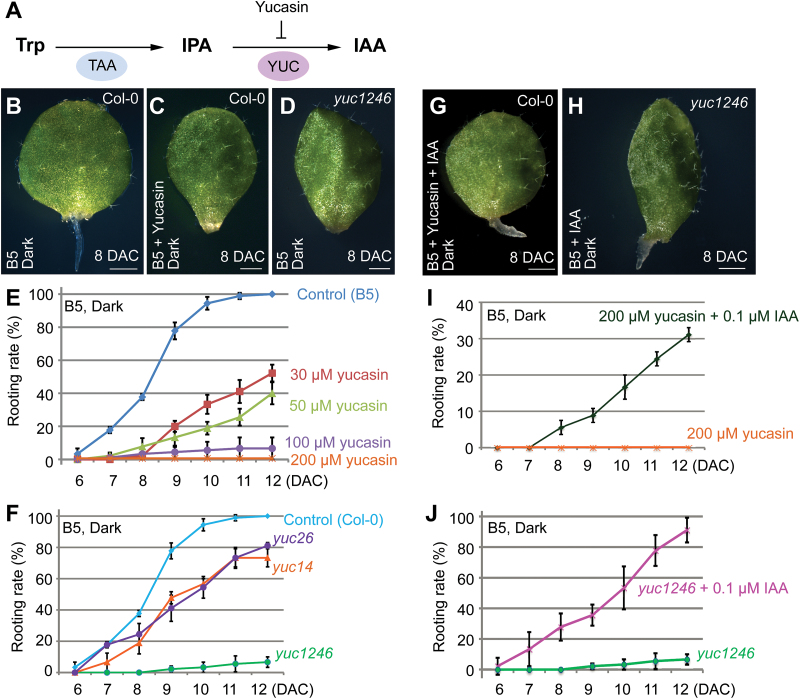
*YUC* is required for *de novo* root organogenesis. (**A**) Yucasin inhibits the YUC protein function in the auxin biogenesis pathway. (**B, C**) Leaf explants cultured on B5 medium (B) or B5 medium with 200 μM yucasin (C) at 8 DAC. Note that yucasin is sufficient to block rooting. (**D**) Leaf explants from *yuc1246* mutant on B5 medium, showing rooting defect at 8 DAC. (**E**) Rooting rate of leaf explants (percentage of leaf explants with regenerated adventitious roots) on B5 medium with different concentration of yucasin treatment. (**F**) Rooting rate of leaf explants from wild-type Col-0, *yuc14* double mutant, *yuc26* double mutant, and *yuc1246* quadruple mutant on B5 medium. This experiment was performed together with the yucasin treatment experiment in E, therefore using the same control data. (**G**) Addition of 0.1 μM IAA partially eliminated the rooting defects caused by 200 μM yucasin treatment. (**H**) Addition of 0.1 μM IAA eliminated the rooting defects caused by simultaneous mutations in *YUC1*, *YUC2*, *YUC4*, and *YUC6*. (**I**) Rooting rate of leaf explants on B5 medium with 200 μM yucasin and 0.1 μM IAA treatment. This experiment was performed together with the yucasin treatment experiment in E, therefore using the same data for 200 μM yucasin treatment. (**J**) Rooting rate of leaf explants from *yuc1246* quadruple mutant on B5 medium with 0.1 μM IAA treatment. This experiment was performed together with the experiment in F, therefore using the same data for *yuc1246*. Bars in E, F, I, and J show SD with three biological replicates. *n* = 30 in each replicate. Scale bars, 1mm in B–D, G, and H.

To study whether *YUC*-mediated auxin biogenesis is required for rooting from leaf explants, we used an auxin biosynthesis inhibitor, yucasin, which specifically inhibits the function of YUC proteins (Fig. 3A) (Nishimura *et al.*, 2014) and thereby reduces auxin levels during regeneration on B5 medium (see Supplementary Fig. S1 at *JXB* online). The wild-type leaf explants started regenerating adventitious roots by 6–8 DAC on B5 medium (Fig. 3B, E). By contrast, regeneration was blocked when yucasin was supplemented in B5 medium (Fig. 3C, E). To confirm this result, we tested the rooting ability in *yuc1 yuc2 yuc4 yuc6* (called *yuc1246*) quadruple mutants, *yuc1 yuc4* (called *yuc14*) double mutants, and *yuc2 yuc6* (called *yuc26*) double mutants. Our results showed that rooting was partially blocked in *yuc14* and *yuc26*, and was severely blocked in *yuc1246* (Fig. 3D, F).

To confirm that the rooting defect was caused by the reduced endogenous auxin production as a result of yucasin treatment, we added a low concentration of IAA (0.1 μM) to the yucasin-containing B5 medium. The addition of IAA partially eliminated the rooting defects caused by yucasin (Fig. 3G, I). Likewise, the addition of IAA also eliminated the rooting defects caused by *YUC* mutations (Fig. 3H, J), suggesting that the phenotype of rooting defect is caused by the lack of auxin in leaf explants of *yuc1246* mutants and is unrelated to the developmental defects of *yuc1246* leaves.

The data from *yuc* mutants, together with yucasin treatment, reveal that *YUC*-mediated auxin biosynthesis is critical for *de novo* root organogenesis.

### *YUC*-mediated auxin biosynthesis acts upstream to cell fate transition

**Fig. 4.**
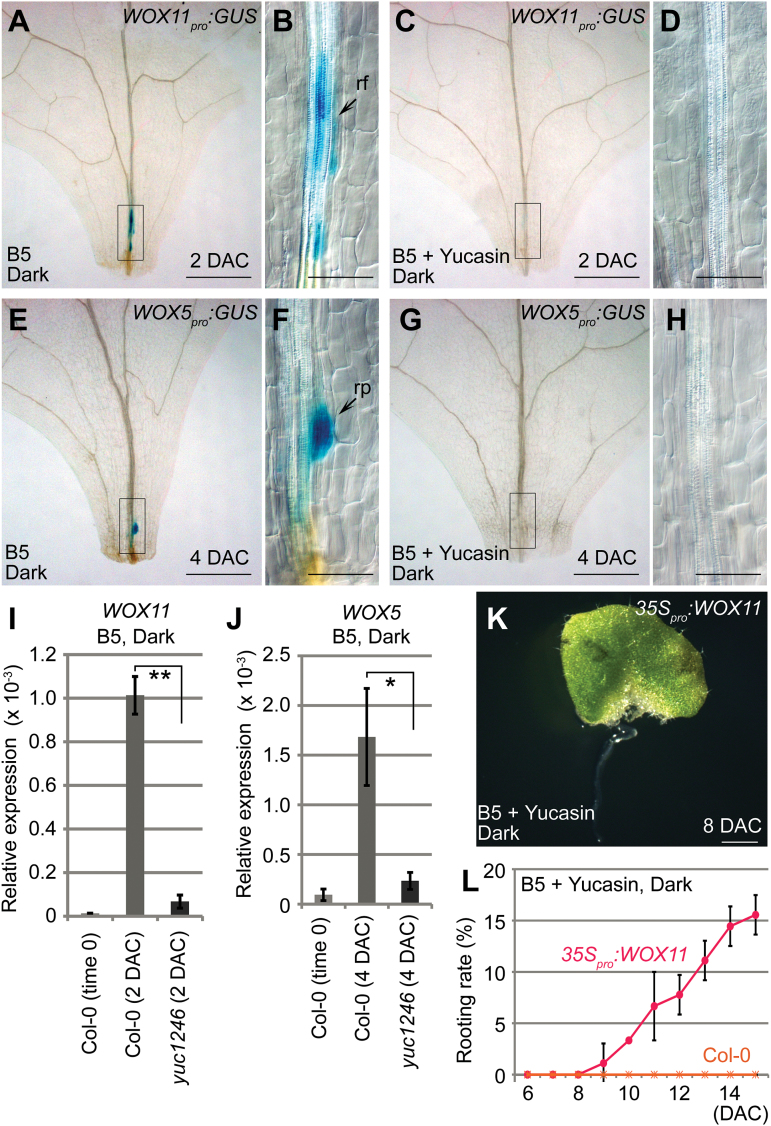
Auxin biogenesis is required for the first step of cell fate transition. (**A–D**) GUS staining of 2-DAC leaf explants from *WOX11*_*pro*_ *:GUS* cultured on B5 medium (A and B) and B5 medium with 200 μM yucasin (C and D). (**E–H**) GUS staining of 4-DAC leaf explants from *WOX5*_*pro*_ *:GUS* cultured on B5 medium (E and F) and B5 medium with 200 μM yucasin (G and H). (**I, J**) qRT-PCR analysis of *WOX11* (I) and *WOX5* (J) in wild type and *yuc1246* mutant leaf explants on B5 medium. Bars show SEM with three biological replicate. Each biological replicate was performed with three technical replicates. *P < 0.05 and **P < 0.01 in two-sample *t* tests. (**K**) Overexpression of *WOX11* partially reverses the rooting defects caused by 200 μM yucasin treatment. (**L**) Rooting rate of leaf explants from wild-type Col-0 and *35S*_*pro*_ *:WOX11* on B5 medium with 200 μM yucasin treatment. Bars show SD with three biological replicates. *n* = 30 in each repeat. B, D, F, and H are close-up views of the boxed regions in A, C, E, and G, respectively. Scale bars, 500 μm in A, C, E, G; 100 μm in B, D, F, H; and 1mm in K.

To identify the step in which auxin biosynthesis functions, we used two marker genes, *WOX11* and *WOX5,* to monitor two steps of cell fate transition. In wild-type leaf explants, expression of *WOX11* appeared around 2 DAC, suggesting the completion of the first step of cell fate transition from competent cells to root founder cells (Fig. 4A, B) (Liu *et al.*, 2014). Expression of *WOX5* was observed at around 4 DAC when the second step of cell fate transition from root founder cells to root primordium cells was completed (Fig. 4E, F) (Liu *et al.*, 2014). By contrast, when yucasin was added to B5 medium to inhibit auxin biosynthesis, the expression of neither *WOX11* nor *WOX5* was observed in leaf explants during culture (Fig. 4C, D, G, H), suggesting that auxin biosynthesis acts upstream to the first step of cell fate transition. Because auxin production was only partially blocked by yucasin treatment (see Supplementary Fig. S1 at *JXB* online), it is possible that a certain auxin concentration is required to induce *WOX11* expression. In addition, qRT-PCR analysis of *WOX11* and *WOX5* expression during the process of rooting confirmed that the first step of cell fate transition was severely blocked in the *yuc1246* mutant (Fig. 4I, J).

To test whether blocking the first step of cell fate transition is a major downstream event caused by a defect in *YUC*-mediated auxin biosynthesis, we overexpressed the *WOX11* gene (see Supplementary Fig. S2 at *JXB* online) in leaf explants cultured on B5 medium supplemented with yucasin. Our results showed that overexpression of *WOX11* was able to partially eliminate the regeneration defects caused by yucasin (Fig. 4K, L), suggesting that *WOX11*-mediated cell fate transition is a target of auxin in *de novo* root organogenesis.

### *YUC4* and *YUC1* are dynamically expressed in mesophyll and competent cells in response to wounding

**Fig. 5.**
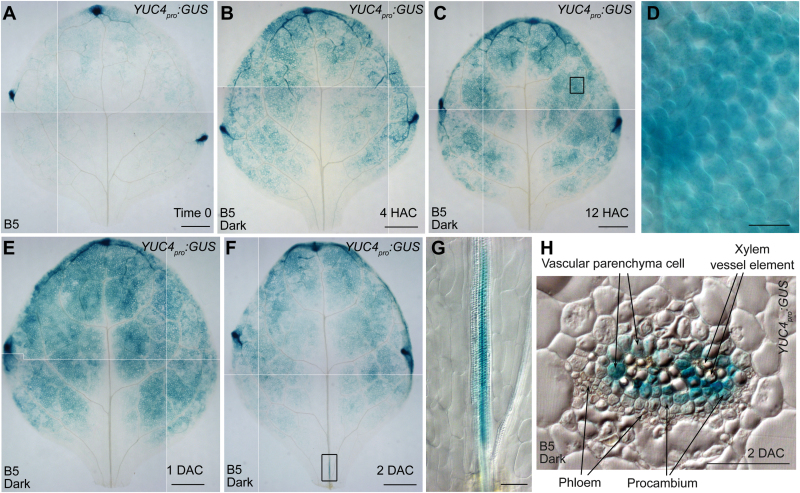
Spatial expression patterns of *YUC4*. (**A–C**) GUS staining of time-0 (A), 4-HAC (B), and 12-HAC (C) leaf explants from *YUC4*_*pro*_ *:GUS* cultured on B5 medium. (**D**) Close-up of the boxed regions in C, showing *YUC4* expression in mesophyll. (**E, F**) GUS staining of 1-DAC (E) and 2-DAC (F) leaf explants from *YUC4*_*pro*_ *:GUS* cultured on B5 medium. (**G**) Close-up of the boxed regions in F, showing *YUC4* expression in the vasculature near a wound. (**H**) Thin sectioning of 2-DAC leaf explants from *YUC4*_*pro*_ *:GUS* cultured on B5 medium at the leaf base, showing the midrib of vasculature as indicated in G. Note that the GUS signal was shown in competent cells. The data in A, B, C, E, and F were pasted together from small pictures of the same leaf explant because the microscope was unable to capture the entire leaf explant in a single visual field. Scale bars, 500 μm in A, B, C, E, F; and 50 μm in D, G, H.

To further analyse the spatial expression patterns of *YUC* genes, we constructed the *YUC4*_*pro*_ *:GUS* and *YUC1*_*pro*_ *:GUS* transgenic reporter lines to monitor the activities of their promoters. In leaf explants of *YUC4*_*pro*_ *:GUS*, there was a low GUS signal in mesophyll cells and relatively strong expression in the hydathode at time 0 (Fig. 5A). During regeneration, the GUS signal became elevated in mesophyll cells at 4 and 12 HAC (Fig. 5B–D), and continued to climb at 1 DAC (Fig. 5E). Interestingly, we also observed a GUS signal of *YUC4*_*pro*_ *:GUS* in the vasculature near the wound at 2 DAC (Fig. 5F, G). Therefore, *YUC4* is expressed in mesophyll cells prior to its expression in vasculature during regeneration.

To further examine the expression of *YUC4* in the vasculature, we performed thin sectioning to show the GUS signals of *YUC4*_*pro*_ *:GUS* at 2 DAC. Our data showed that *YUC4* was expressed in the procambium and vascular parenchyma cells (Fig. 5H), which serve as competent cells during regeneration (Liu *et al.*, 2014).

Compared with *YUC4*_*pro*_ *:GUS*, there was a weak GUS signal in *YUC1*_*pro*_ *:GUS*. The GUS signal from *YUC1*_*pro*_ *:GUS* was barely detected in time-0 leaf explants, and was elevated in mesophyll cells as well as the vasculature near the wound at 1 DAC (see Supplementary Fig. S3 at *JXB* online).

**Fig. 6.**
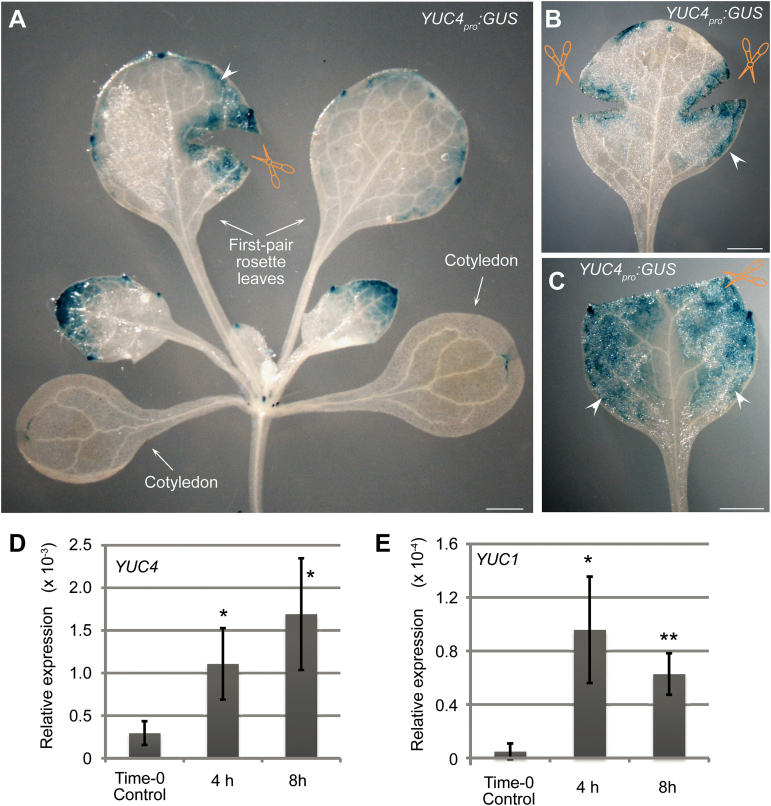
*YUC4* and *YUC1* were induced by wounding. (**A–C**) GUS staining of wounded leaves from *YUC4*_*pro*_ *:GUS* at 4 hours after wounding, showing one (A) and two (B) wounds, or removal of the leaf tip (C). Arrowheads indicate the GUS signal that spreads in the mesophyll. (**D, E**) qRT-PCR analysis of *YUC4* (D) and *YUC1* (E) expression levels in wounded leaves. The tissue materials for RNA extraction are from attached leaves with one wound as indicated in A. Bars show SEM from three biological replicates. Each biological replicate was performed with three technical replicates. *P < 0.05 and **P < 0.01 in two-sample *t* tests compared with time-0 control. Scale bars, 1mm in A–C.

In addition, we showed that the expression of *YUC4* and *YUC1* was induced by wounding. The GUS signal of *YUC4*_*pro*_ *:GUS* was observed in mesophyll cells in and near the wounded region of leaves that were not detached from the plant (Fig. 6A–C; for control, see Supplementary Fig. S4 at *JXB* online). A GUS signal was also observed in the mesophyll that was not near the wound (arrowheads in Fig. 6A–C; for control, see Supplementary Fig. S4), suggesting that the wound signal that triggers *YUC* expression is able to spread within leaves. The qRT-PCR analysis showed that the expression levels of *YUC4* and *YUC1* were quickly induced in the attached leaves within 8 hours after wounding (Fig. 6D, E). These data indicate that the previously proposed wound signal (Liu *et al.*, 2014) may have an effect on the upregulation of expression of the two *YUC* genes.

### *YUC5*, *YUC8*, and *YUC9* act primarily in response to darkness

**Fig. 7.**
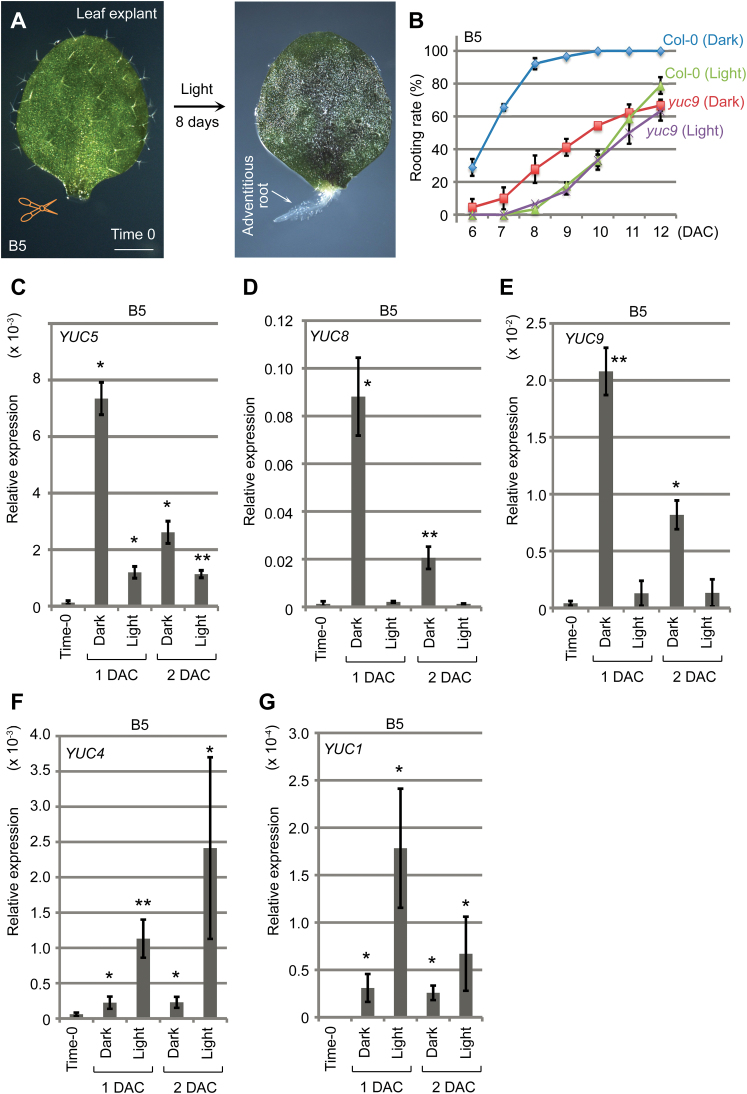
*YUC* expression during regeneration in dark and light. (**A**) *De novo* root organogenesis on B5 medium without sucrose in light. (**B**) Rooting rate of leaf explants from wild-type Col-0 and *yuc9* on B5 medium in light and dark conditions. Bars show SD with three biological replicates. *n* = 30 in each repeat. (**C–G**) qRT-PCR analysis of *YUC5* (C), *YUC8* (D), *YUC9* (E), *YUC4* (F), and *YUC1* (G) expression during regeneration on B5 medium in either dark or light conditions. Bars show SEM from three biological replicates. Each biological replicate was performed with three technical replicates. *P < 0.05 and **P < 0.01 in two-sample *t* tests compared with time-0 control.

Regeneration of explants in natural conditions may occur in either dark or light conditions. The regeneration system described above was in dark conditions. We also tested regeneration of adventitious roots in light conditions by culturing leaf explants on B5 medium without sucrose (Fig. 7A) (Chen *et al.*, 2014). The rooting rate of wild-type leaf explants was slower in light conditions than that in dark conditions (Fig. 7B). The auxin level was also elevated in wild-type leaf explants cultured in light conditions during regeneration (see Supplementary Fig. S5 at *JXB* online). However, increase in auxin levels in leaf explants cultured in light conditions was lower than that in leaf explants cultured in dark conditions (Supplementary Fig. S5 compared with Fig. 1B–G). Therefore, leaf explants produce more auxin and have a higher regenerative efficiency in the dark.

**Fig. 8.**
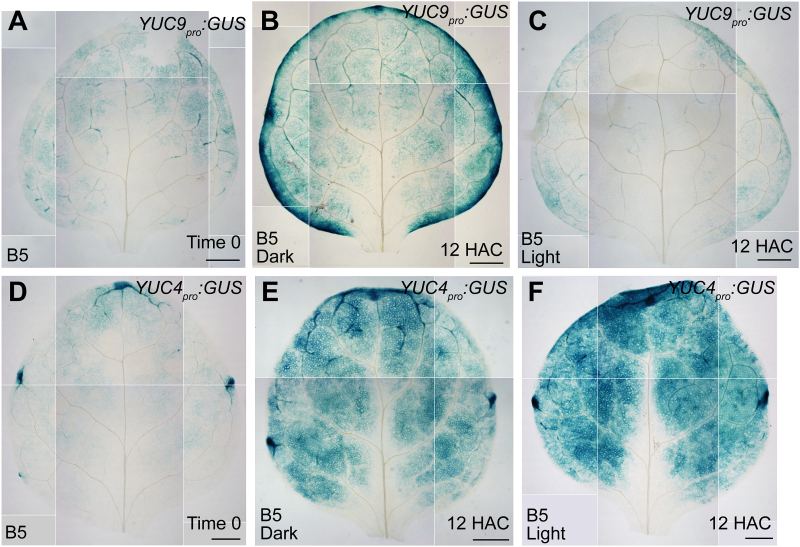
Expression patterns of *YUC9* and *YUC4* in dark and light conditions. (**A**) GUS staining of the time-0 leaf explant from *YUC9*_*pro*_ *:GUS*. (**B, C**) GUS staining of 12-HAC leaf explants from *YUC9*_*pro*_ *:GUS* cultured on B5 medium in dark (B) or light (C) conditions. (**D**) GUS staining of the time-0 leaf explant from *YUC4*_*pro*_ *:GUS*. (**E, F**) GUS staining of 12-HAC leaf explants from *YUC4*_*pro*_ *:GUS* cultured on B5 medium in dark (E) or light (F) conditions. The data in A–F were pasted together from small pictures of the same leaf explant because the microscope was unable to capture the entire leaf explant in a single visual field. Scale bars, 500 μm in A–F.

Previous studies revealed that the expression of *YUC5*, *YUC8*, and *YUC9* is upregulated by dark treatment (Tao *et al.*, 2008; Hornitschek *et al.*, 2012); therefore, they could contribute to the regeneration system in darkness. We performed qRT-PCR analysis of the three closely related *YUC* genes (see Supplementary Fig. S6 at *JXB* online) in both dark and light conditions. The results showed that expression of *YUC5*, *YUC8*, and *YUC9* was dramatically elevated during regeneration in dark conditions within 2 DAC (Fig. 7C–E). Expression of *YUC5* was also elevated in light conditions during regeneration, but its increase was much lower in light conditions than in dark conditions (Fig. 7C). To analyse the spatial expression pattern of *YUC9*, we constructed the *YUC9*_*pro*_ *:GUS* transgenic reporter line. In leaf explants of *YUC9*_*pro*_ *:GUS*, the GUS signal became elevated in leaf margin and mesophyll cells at 12 HAC in dark conditions compared with that at time 0 (Fig. 8A, B). In contrast, we did not observe an increase in GUS signal at 12 HAC when leaf explants of *YUC9*_*pro*_ *:GUS* were cultured in light conditions (Fig. 8C compared with Fig. 8A, B).

We also analysed the rooting ability of the *yuc9* mutant. The result showed that rooting was partially defective in *yuc9* leaf explants compared with that in wild-type leaf explants when they were cultured in dark conditions (Fig. 7B). In contrast, we did not observe a clear rooting defect of *yuc9* leaf explants compared with the rooting ability of wild-type leaf explants when they were cultured in light conditions (Fig. 7B).

The data from *YUC5*, *YUC8*, and *YUC9* suggest that the three genes may act predominantly in response to darkness but might also have a minor role in response to wounding as indicated by the previous study (Hornitschek *et al.*, 2012).

Expression of *YUC4* and *YUC1* was elevated during regeneration in both light and dark conditions, although the two genes had a more severely elevated level of expression in light than in dark within 2 DAC (Fig. 7F, G). This might occur because of the low expression levels of *YUC5*, *YUC8*, and *YUC9* in light, which may result in feedback that stimulates the expression of *YUC4* and *YUC1* for regeneration. In addition, in leaf explants of *YUC4*_*pro*_ *:GUS*, the GUS signal became elevated in mesophyll cells at 12 HAC compared with the signal at time 0 in both dark and light conditions (Fig. 8D–F). Because sucrose was not added to the B5 medium when leaf explants were cultured in light conditions, the elevated *YUC4* expression level could not have been related to the presence of sucrose in the medium (Fig. 8F). The data from *YUC4* and *YUC1* suggest that the two genes may act predominantly in response to wounding, regardless of light and dark conditions.

## Discussion

*De novo* root organogenesis commonly occurs when plant organs are detached or wounded, and auxin is known to have an important role in this process ( Greenwood *et al.*, 2001; De Klerk, 2002; Ahkami *et al.*, 2009; Correa Lda *et al.*, 2012; Chen *et al.*, 2014; Liu *et al.*, 2014; Xu and Huang, 2014). In this study, we show that *YUC*-mediated *de novo* synthesis of auxin is enhanced after detachment of leaf explants, and this in turn is required for *de novo* root organogenesis from leaf explants.

**Fig. 9.**
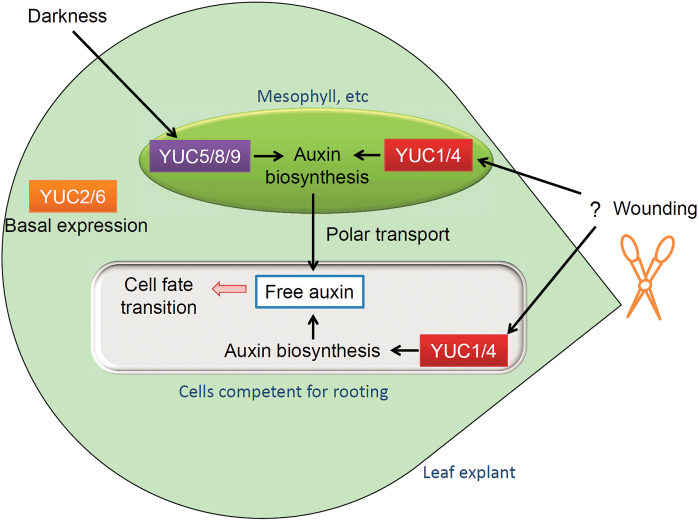
Model for *YUC*-mediated auxin biogenesis in *de novo* root organogenesis. *YUC1*/*4*-mediated auxin biogenesis produces auxin in both mesophyll cells and competent cells primarily in response to wounding. The wound signal that induces *YUC1*/*4* expression in mesophyll cells is not clear (question mark). *YUC5*/*8*/*9*-mediated auxin biogenesis produces auxin in mesophyll cells primarily in response to darkness. *YUC2*/*6*-mediated auxin biogenesis contributes to the basal auxin level in the leaf and also contributes to regeneration.

The Arabidopsis genome encodes at least 11 *YUC* genes (see Supplementary Fig. S6 at *JXB* online) (Cheng *et al.*, 2006), and our data showed that mutations in *YUC1*, *YUC2*, *YUC4*, and *YUC6* severely block rooting from leaf explants, suggesting that these four genes have primary roles in regeneration among all the YUC family. However, expression of *YUC2* and *YUC6* was not significantly induced in leaf explants within 2 DAC (see Supplementary Fig. S7 at *JXB* online) and these two gene loci are not regulated by the H3K27me3 epigenetic marker (He *et al.*, 2012); this suggests that these two genes may contribute to the basal auxin level in the leaf. In contrast to *YUC2* and *YUC6*, *YUC4* and *YUC1* were induced by wounding. We propose that both basally expressed *YUC2* and *YUC6* and wounding-induced *YUC4* and *YUC1* contribute to the auxin level during rooting from leaf explants, but that it is *YUC4* and *YUC1* that primarily contribute to the rapid increase in auxin in leaf explants after detachment (see the model in Fig. 9). This may explain the phenotype analysis that *yuc14* and *yuc26* are partially defective in rooting whereas rooting in *yuc1246* is severely blocked.

The expression patterns of *YUC4* and *YUC1* suggest that they have dual roles (see the model in Fig. 9). First, they rapidly produce auxin in mesophyll cells within and after 4 HAC, and the auxin is then polar transported toward competent cells near the wound (Liu *et al.*, 2014). Second, they produce auxin in competent cells near the wound around 1–2 DAC when the cell fate transition is occurring. We propose that the first role of *YUC* (auxin production in mesophyll cells) is to increase the total auxin level in leaf explants and that some of this auxin is then polar transported to competent cells near the wound, triggering *WOX11* expression and the first step of cell fate transition. This supposition is based on the fact that inhibition of polar auxin transport from the mesophyll to the vasculature severely blocks rooting (Liu *et al.*, 2014). The second role of *YUC* (auxin production in competent cells) might involve the maintenance of the maximum auxin level in competent cells, because this occurs later than auxin production in the mesophyll.

The auxin production levels in leaf explants are different in dark and light conditions. Expression levels of *YUC4* and *YUC1* were upregulated in both dark and light conditions in leaf explants, and *yuc1246* had defective rooting in both dark conditions (Fig. 3F) and light conditions (data not shown). Therefore, *YUC1*, *YUC2*, *YUC4*, and *YUC6* contribute to rooting regardless of whether the leaves are in light or dark conditions. In contrast, induction of *YUC5*, *YUC8*, and *YUC9* expression was primarily in response to darkness, and *yuc9* has a rooting defect in the dark but not in the light. Therefore, the major role of *YUC5*, *YUC8*, and *YUC9* is to produce auxin in response to darkness (see the model in Fig. 9).

Expression of *YUC10* and *YUC11* was not detected during rooting from leaf explants in our conditions (see Supplementary Fig. S8 at *JXB* online). *YUC3* was expressed in time-0 leaf explants, while its expression level was reduced when leaf explants were cultured on B5 medium (Supplementary Fig. S8). These data indicate that *YUC10*, *YUC11*, and *YUC3* might not be involved in rooting from leaf explants. *YUC7* was expressed in leaf explants during regeneration (Supplementary Fig. S8). However, the role of *YUC7* in regeneration is not clear.

Currently, it remains unclear how wounds induce *YUC1* and *YUC4* expression. Whether *YUC1* and *YUC4* are the direct targets of wounding is also not known. Wounds may produce many physical and chemical signals, and may also induce hormone actions and wounding-sensitive gene expression (Leon *et al.*, 2001; Maffei *et al.*, 2007; Iwase *et al.*, 2011; Xu and Huang, 2014). However, the concept of a ‘wound signal’ is still very poorly understood. It is possible that the factors triggering *YUC1* and *YUC4* expression in leaf explants upon wounding may cooperate with epigenetic factors, such as those in the H3K27me3 modification, because our data suggested that upregulation of *YUC* genes is accompanied by the removal of H3K27me3. Gaining an understanding the complex wound signal and epigenetic regulation of *YUC* genes will improve our knowledge of the mechanisms that occur at the very start of regeneration.

## Supplementary data

Supplementary data are available at *JXB* online.

Figure S1. Yucasin inhibits auxin production in regeneration.

Figure S2. *WOX11* expression in *35S*_*pro*_ *:WOX11* transgenic plants.

Figure S3. Spatial expression patterns of *YUC1* in regeneration.

Figure S4. Expression of *WOX5* is not in response to wounding within 4h.

Figure S5. Auxin production in leaf explants in light conditions.

Figure S6. YUC family in Arabidopsis.

Figure S7. Expression of *YUC2* and *YUC6* genes in regeneration.

Figure S8. *YUC3*, *YUC7*, *YUC10*, *YUC11*, and *YUC9* expression during rooting from leaf explants.

Table S1. List of primers used in this study.

Supplementary Data
