# Supplementary material for: YUCCA-mediated auxin biogenesis is required for cell fate transition occurring during de novo root organogenesis in Arabidopsis
Source: J Exp Bot. 2016 Jun 2;67(14):4273–84. doi: 10.1093/jxb/erw213 (PMC5301932; doi:10.1093/jxb/erw213)
Supplement: Supplementary Data [file supp_67_14_4273__index.html]

 YUCCA-mediated auxin biogenesis is required for cell fate transition occurring during de novo root organogenesis in Arabidopsis — Supplementary Data 

# *YUCCA*-mediated auxin biogenesis is required for cell fate transition occurring during *de novo* root organogenesis in Arabidopsis

## Supplementary Data

Data files

- supplementary\_figures\_S1\_S8\_table\_S1.pdf - Supplementary Data
