# Supplementary material for: YUCCA-mediated auxin biogenesis is required for cell fate transition occurring during de novo root organogenesis in Arabidopsis
Source: J Exp Bot. 2016 Jun 2;67(14):4273–84. doi: 10.1093/jxb/erw213 (PMC5301932; doi:10.1093/jxb/erw213)
Supplement: Supplementary Data [file supp_erw213_supplementary_figures_S1_S8_table_S1.pdf]

SUPPLEMENTARY DATA

YUCCA-mediated auxin biogenesis is required for cell fate transition occurring during *de novo* root organogenesis in *Arabidopsis*

Lyuqin Chen[1,2], Jianhua Tong[3], Langtao Xiao[3], Ying Ruan[3], Jingchun Liu[1, 4, *], Minhuan Zeng[1], Hai Huang[1], Jia-Wei Wang[1], and Lin Xu[1, #]

1, National Key Laboratory of Plant Molecular Genetics, CAS Center for Excellence in Molecular Plant Sciences, Institute of Plant Physiology and Ecology, Shanghai Institutes for Biological Sciences, Chinese Academy of Sciences, 300 Fenglin Road, Shanghai 200032, China.

2, University of Chinese Academy of Sciences, 19A Yuquan Road, Beijing, 100049, China

3, Pre-National Laboratory for Crop Germplasm Innovation and Resource Utilization, Hunan Agricultural University, Changsha, Hunan 410128, China

4, College of Life and Environment Sciences, Shanghai Normal University, Shanghai 200234, China.

*, Present address, Department of Plant and Microbial Biology, Zürich-Basel Plant Science Center, University of Zürich, Zollikerstrasse 107, 8008 Zürich, Switzerland

[#] Author for correspondence
E-mail: xulin01@sibs.ac.cn

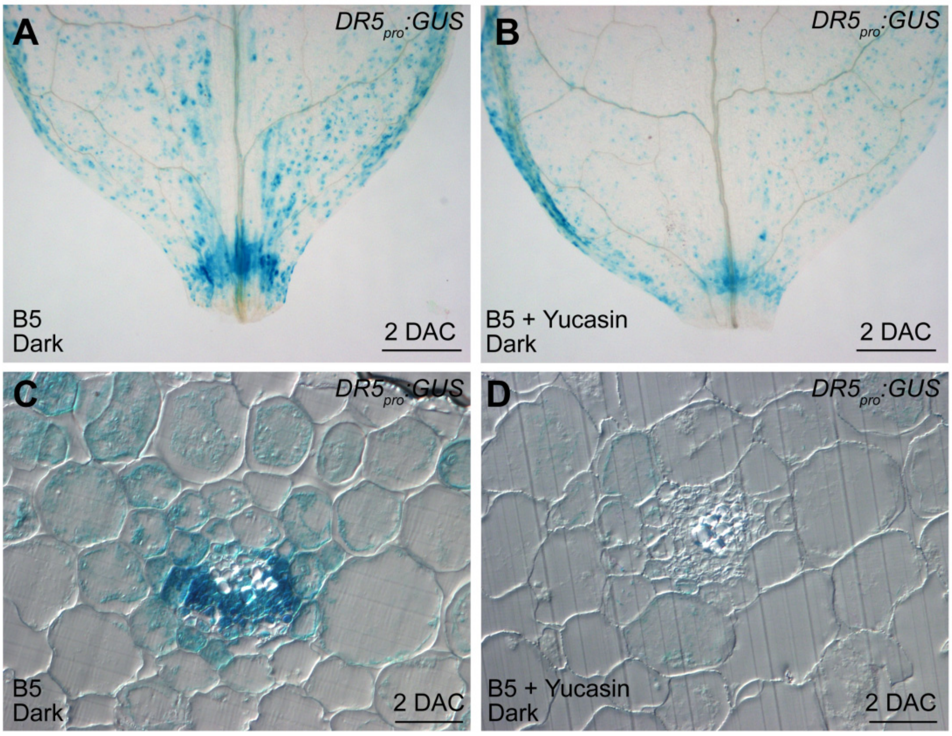

Fig. S1. Yucasin inhibits auxin production in regeneration.

(**A, B**) Observations of the GUS signal in leaf explants from *DR5*$_{pro}$*:GUS* reporter line at 2 DAC cultured on B5 medium (A) or B5 medium with 200 µM yucasin treatment (B).

(**C, D**) Thin sectioning of 2-DAC leaf explants from *DR5*$_{pro}$*:GUS* cultured on B5 medium (C) or B5 medium with 200 µM yucasin treatment (D) at the leaf base, showing the midrib of vasculature.

Note that the auxin level was lower in the vasculature of wounded region of leaf explants cultured on B5 medium with 200 µM yucasin treatment compared with that in leaf explants cultured on B5 medium. Yucasin treatment could not strictly block all YUC proteins in the whole leaf explant. However, the partially reduced auxin level in the wounding site is sufficient to block rooting.

Scale bars, 500 µm in A, B; and 50 µm in C, D.

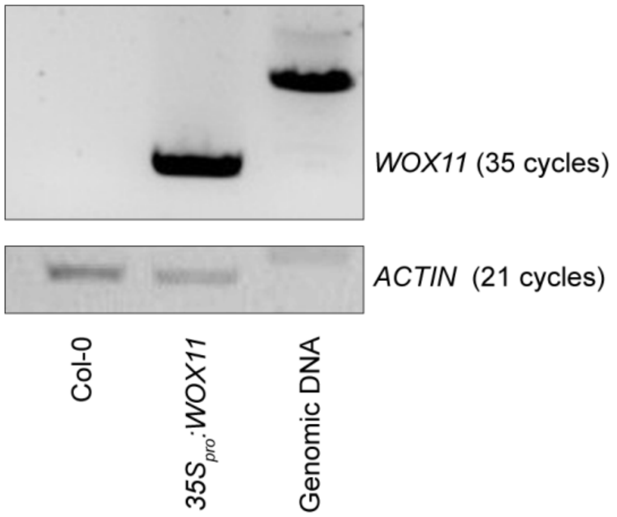

Fig. S2. *WOX11* expression in *35S$_{pro}$:WOX11* transgenic plants.

RT-PCR analysis of *WOX11* expression in time-0 leaf explants from Col-0 and *35S$_{pro}$:WOX11* transgenic plants. Expression of *ACTIN* was served as an internal control.

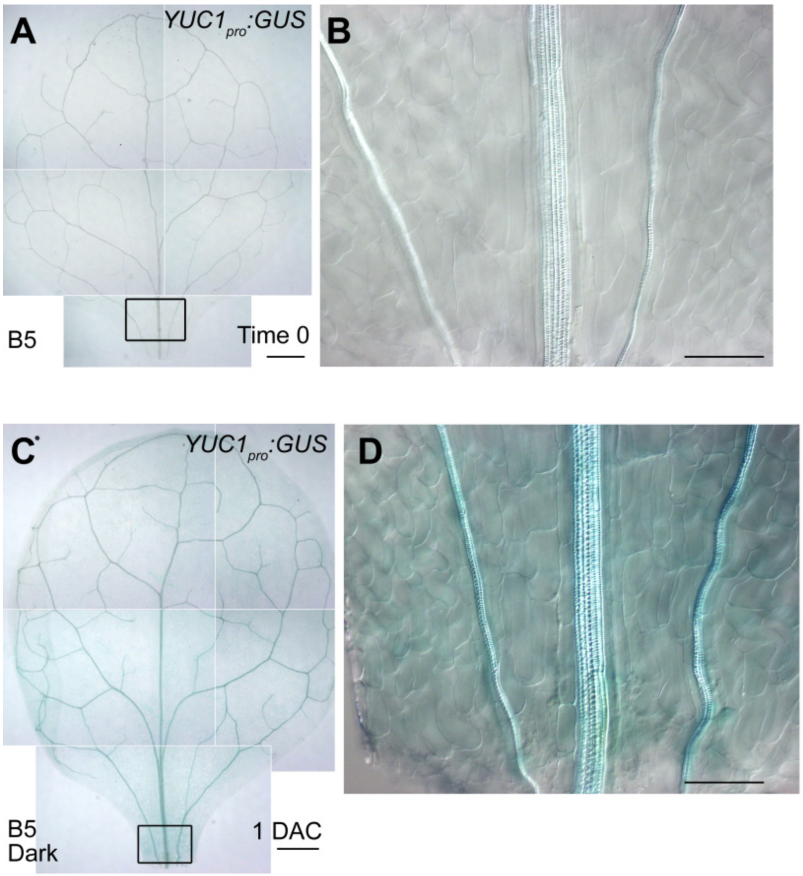

Fig. S3. Spatial expression patterns of *YUC1* in regeneration.

(**A–D**) GUS staining of time-0 (A, B) and 1-DAC (C, D) leaf explants from *YUC1*$_{pro}$*:GUS* cultured on B5 medium.

The data in A and C were pasted by small pictures of the same leaf explant, because the microscopy is unable to capture the entire leaf explants at a single visual field. B and D are close-ups of the boxed regions in A and C, respectively. Scale bars, 500 µm in A, C; and 50 µm in B, D.

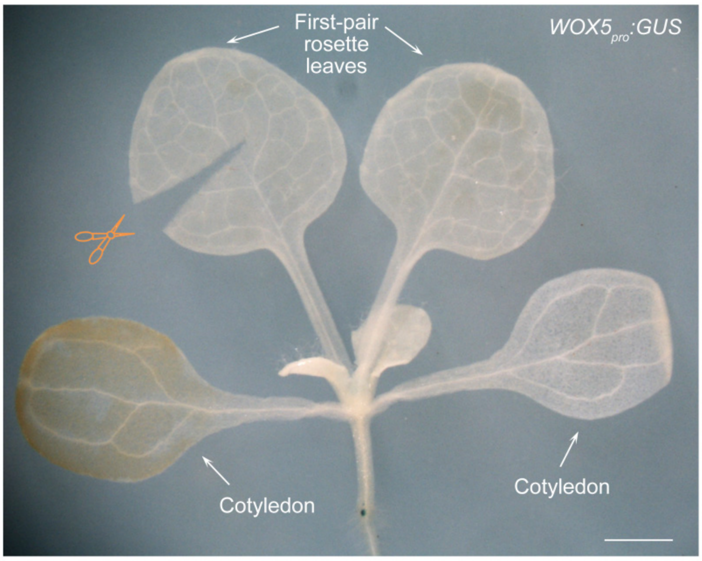

Fig. S4. Expression of *WOX5* is not in response to wounding within 4 h.

GUS staining of a wounded leaf from $WOX5_{pro}$:*GUS* at 4 hours after wounding, serving as a negative control for non-specific GUS staining in Fig. 6A–C. Note that GUS signal was not observed at the wounded site. Scale bar, 1 mm.

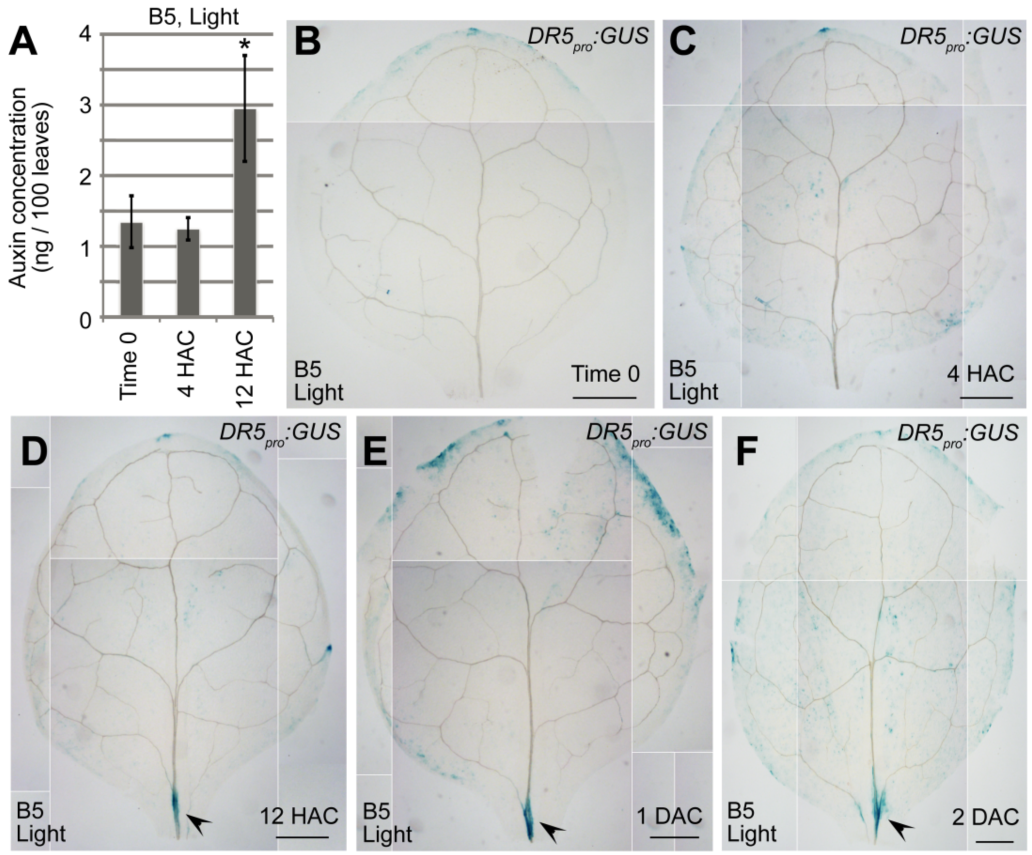

Fig. S5. Auxin production in leaf explants in light conditions.

(**A**) Auxin concentration in leaf explants from time 0 to 12 HAC on B5 medium in light conditions. Bars show SEM with three biological repetitions. Each biological repetition was performed with three technical repetitions. * $P < 0.05$ in two-sample *t* tests compared with time-0 control.

(**B–F**) Observations of the GUS signal in leaf explants from *DR5*$_{pro}$*:GUS* reporter line at time 0 (B), 4 HAC (C), 12 HAC (D), 1 DAC (E) and 2 DAC (F) cultured in light conditions. Arrowheads in D–F indicate the GUS signal in vasculature near the wound.

The data in B–F were pasted by small pictures of the same leaf explant, because the microscopy is unable to capture the entire leaf explants at a single visual field. Scale bars, 500 μm in B–F.

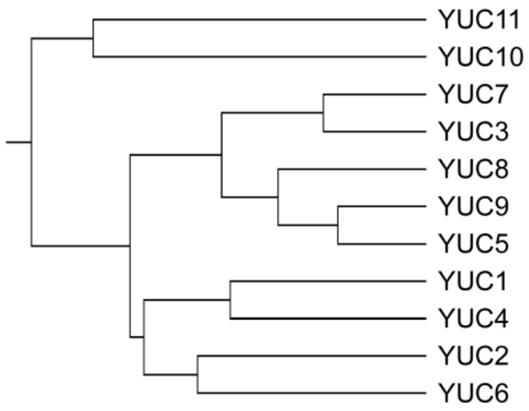

Fig. S6. YUC family in Arabidopsis.

Phylogenetic analysis of *Arabidopsis* YUC protein sequences was conducted using MEGA3.0 (Kumar *et al.*, 2004).

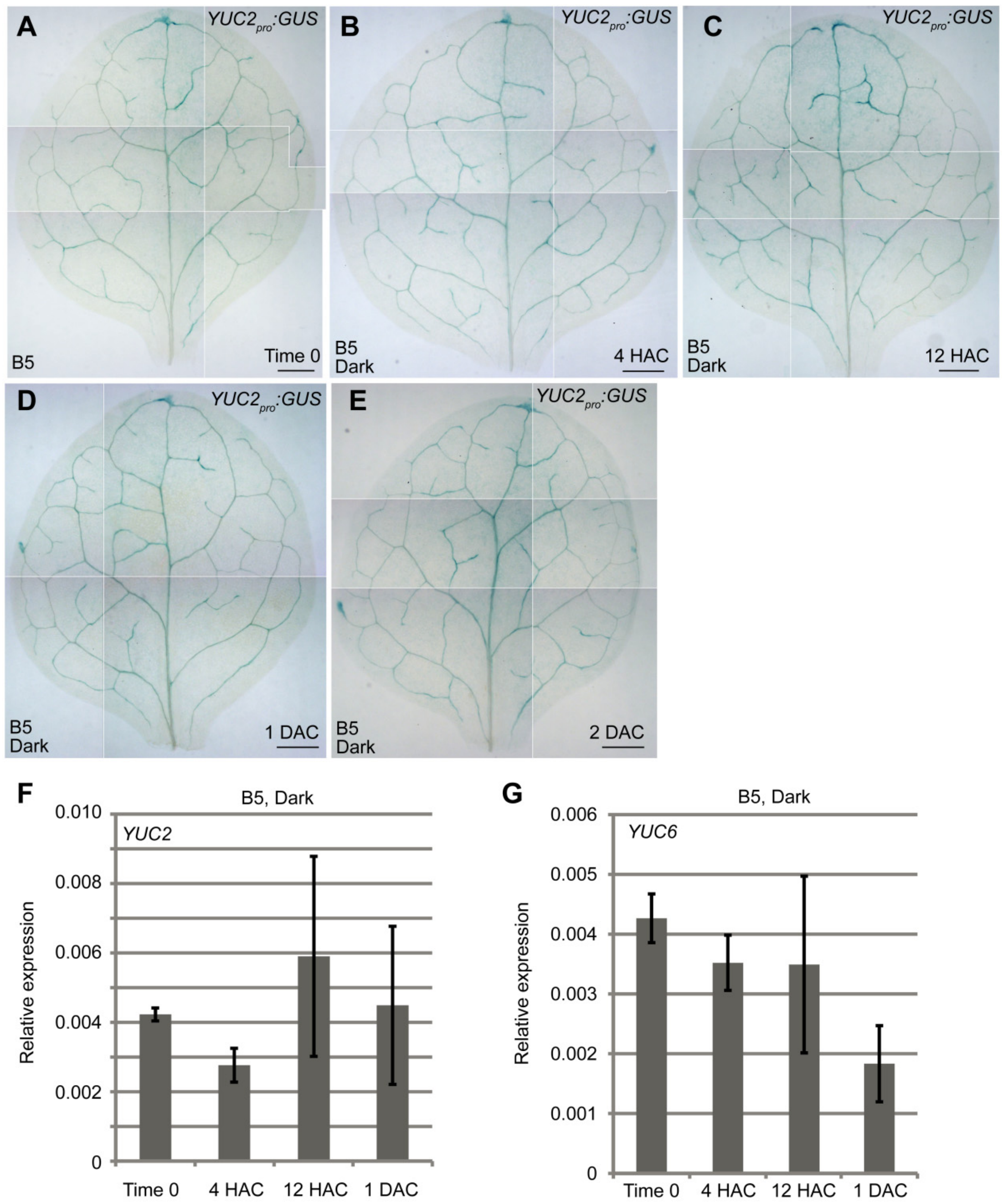

Fig. S7. Expression of *YUC2* and *YUC6* genes in regeneration.

(**A–E**) GUS staining of time-0 (A), 4-HAC (B), 12-HAC (C), 1-DAC (D) and 2-DAC (E) leaf explants from *YUC2*$_{pro}$*:GUS* cultured on B5 medium. The data in A–E were pasted by small pictures of the same leaf explant, because the microscopy is unable to capture the entire leaf explants at a single visual field.

(**F, G**) qRT-PCR analysis of *YUC2* (F) and *YUC6* (G) during rooting from leaf explants on B5 medium. Bars show SEM from three biological repetitions. Each biological repetition was performed with three technical repetitions. Note that the

expression levels of the two genes were not significantly upregulated within 1 DAC. We also constructed *YUC6*$_{pro}$*:GUS* construct; however, this construct did not show GUS signal in leaf explants in our conditions. Scale bars, 500 µm in A–E.

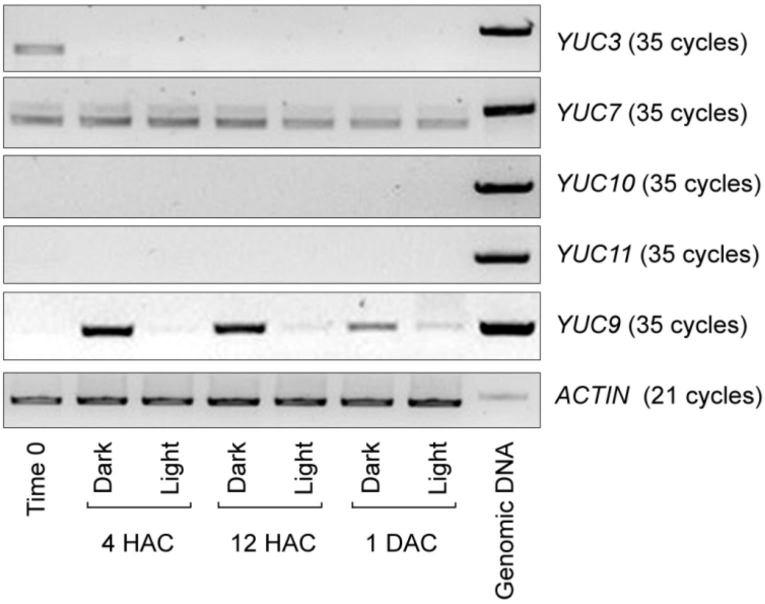

Fig. S8. *YUC3*, *YUC7*, *YUC10*, *YUC11*, and *YUC9* expression during rooting from leaf explants.

RT-PCR analysis of *YUC* gene expression in time-0, 4-HAC, 12-HAC and 1-DAC leaf explants from Col-0 in dark or light conditions. *YUC9* was served as a positive control. Expression of *ACTIN* was served as an internal control.

Table S1. List of primers used in this study.

Experiments	Primers	Sequence (5' →3')
**Molecular cloning**		
YUC4$_{pro}$:GUS	YUC4$_{pro}$-F1	cgcggatccGAAGCCGTTGATTCTTACATGGTG
	YUC4$_{pro}$-R1	tcccccgggGTCGACTAATAAAAGCGAAAG
YUC1$_{pro}$:GUS	YUC1$_{pro}$-F1	cttgcatgcctgcaggtcgacCACGTTTTTGGGTGGACCAC
	YUC1$_{pro}$-R1	cggggatcctctagagtcgacTCTTGATGGATGATGGAAAA
YUC2$_{pro}$:GUS	YUC2$_{pro}$-F1	acgcgtcgacGCATAATCAAATTTTAGTTAC
	YUC2$_{pro}$-R1	cgggatccACAATGTTGAGGACGAGCCAATGG
YUC9$_{pro}$:GUS	YUC9$_{pro}$-F1	cttgcatgcctgcaggtcgacCCAATTGAAAAAAGTGTTAAAC
	YUC9$_{pro}$-R1	cggggatcctctagagtcgacTTTCTTGAGTGAGTTTTTGAATG
**qRT-PCR**		
YUC1	YUC1-F	CGATGTCGGAGCTATGTCTC
	YUC1-R	CTGTACAAGTTTATTACTTCG
YUC2	YUC2-F	CGGTTAGGGTTAGTTCGACC
	YUC2-R	GAACCTCAATATCCTCAGCG
YUC4	YUC4-F	CCGTTCTTGATGTCGGTGCC
	YUC4-R	AAGGATTTATTGAAATGAAGATG
YUC6	YUC6-F	GGTAGTTAAGCACACGTGTC
	YUC6-R	GGCTAGCGTGCCAACGTCGAG
YUC5	YUC5-F	CCATGATGTTGATGAAGTGG
	YUC5-R	CCAATATCTTGAGCGATG
YUC8	YUC8-F	TGTATGCGGTTGGGTTTACGAGGA
	YUC8-R	CCTTGAGCGTTTCGTGGGTTGTTT
YUC9	YUC9-F	GCTCGGTTCATGTTTTACCG
	YUC9-R	CTTGGCTTCAGGAAAGTGAG
WOX11	WOX11-F	CGCAACCACCAACACTTGTGACC
	WOX11-R	AAGACATCTGTTGCATCACC
WOX5	WOX5-F	GTGAAAGGTCGAAGCTTACG
	WOX5-R	GTACTGGTTATTGCCTCTAGC

ACTIN	ACTIN-F	TGGCATCA(T/C)ACTTTCTACAA
	ACTIN-R	CCACCACT(G/A/T)AGCACAATGTT
**RT-PCR**		
YUC3	YUC3-F	CGTTTGAATTAGGAGTTACG
	YUC3-R	GGTATCCCATCATCGGAG
YUC7	YUC7-F	GGATGTGGAAACTCAGGC
	YUC7-R	GTGGCAAGAATCACTGAATC
YUC10	YUC10-F	GGTCGACACATTGGTGACG
	YUC10-R	CCATCCTTCTTCATCACG
YUC11	YUC11-F	GATCTATCTAAGTGCAACGC
	YUC11-R	GAACAGATCTCCATCATCG
YUC9	YUC9-F	GCTCGGTTCATGTTTTACCG
	YUC9-R	CTTGGCTTCAGGAAAGTGAG
ACTIN	ACTIN-F	TGGCATCA(T/C)ACTTTCTACAA
	ACTIN-R	CCACCACT(G/A/T)AGCACAATGTT
**ChIP**		
YUC1	YUC1-I-F	GTCGTATCTCGGTATGCTATAG
	YUC1-I-R	CGTGAACTTCTGCCGACAG
	YUC1-II-F	GAGTAACTTTACCACTAATCTAC
	YUC1-II-R	CATTGTGCATTGTGTGAGCC
YUC4	YUC4-I-F	CATGAGAGGAGTCGAGTTGC
	YUC4-I-R	CTTGCAAGGCCATGTTAAATGTG
	YUC4-II-F	GCTATTGAATAAGTATCTTTCG
	YUC4-II-R	CTGCATGCATGTATCTCTAGC
AG	AG-F	GGCTTTGGAGCAGCAATCAC
	AG-R	GCAAACCATTTCTACGTTTGC

Note that lower case letters represent additional nucleotides to introduce restriction sites.

SUPPLEMENTARY REFERENCE

Kumar S, Tamura K, Nei M. 2004. MEGA3: Integrated software for Molecular Evolutionary Genetics Analysis and sequence alignment. *Brief Bioinform.* **5**, 150-163.
